# *Shewanella putrefaciens*, an emerging foe from climate change: a case report

**DOI:** 10.1186/s13256-025-05100-w

**Published:** 2025-04-14

**Authors:** Francesca Di Bartolomeo, Riccardo Ligresti, Sofia Pettenuzzo, Teresa Bini, Camilla Tincati, Giulia Carla Marchetti

**Affiliations:** Clinic of Infectious Diseases, San Paolo Hospital, ASST Santi Paolo e Carlo, Department of Health Sciences, University of Milan, Milan, Italy

**Keywords:** *Shewanella putrefaciens*, Soft and skin tissue infections (SSTIs), Fluoroquinolones, Case report

## Abstract

**Background:**

*Shewanella putrefaciens* is a Gram negative, facultatively anerobic bacterium commonly found in aquatic environments and is associated with decomposing organic matter. Although typically nonpathogenic, it has been recognized as an opportunistic pathogen capable of causing rare infections in humans, particularly immunocompromised individuals or those with underlying health conditions.

**Case presentation:**

We report the case of a 74-year-old white Italian female who developed a soft tissue infection after sustaining a leg injury and subsequently bathing in the coastal waters of Valencia, Spain. Despite initial treatment with amoxicillin/clavulanic acid and wound debridement, the infection persisted. Microbiological analysis revealed the presence of *Shewanella putrefaciens* and *Bacteroides fragilis*. The patient required a second-line antibiotic regimen with ciprofloxacin, which successfully resolved the infection, although the patient experienced chronic ankle edema owing to underlying lymphatic insufficiency.

**Conclusion:**

This case underscores several critical considerations: the emerging pathogenic potential of *S*. *putrefaciens*, the implications of environmental antibiotic resistance, and the increased risk of such infections in the context of global warming and rising sea temperatures. With climate change contributing to warmer aquatic environments, the proliferation of marine bacteria, such as *S*. *putrefaciens*, may lead to a growing number of opportunistic infections, emphasizing the need for vigilance in both clinical and environmental health settings.

## Background

*Shewanella putrefaciens* is a Gram negative, nonfermenting, facultatively anerobic, rod-shaped bacterium known for its ability to produce biofilms. It is commonly found in aquatic environments and seafood, including food processing facilities and storage areas. As part of the marine microflora, *S*. *putrefaciens*—like other species in the *Shewanella* genus—is associated with the decomposition of organic matter, such as dead fish and algae, owing to its production of hydrogen sulfide (H₂S) [1]. These bacteria thrive under diverse environmental conditions, including fluctuations in temperature, salinity, barometric pressure, and oxygen levels [2]. They are also capable of reducing various metals and substances, such as nitrate, nitrite, thiosulfate, and trimethylamine-*N*-oxide [3].

Although rare, it can cause opportunistic infections in humans, including skin and soft tissue infections (SSTIs), such as cellulitis and wound-related infections, bacteremia, intra-abdominal infections, and hepatobiliary diseases (owing to the lipophilic nature of the bacterium), as well as otitis. In some cases, it may also lead to joint and bone conditions, such as arthritis and osteomyelitis [2].

Treatment typically requires a combination of antibiotic therapy, guided by antimicrobial susceptibility tests, and local interventions, such as surgical debridement or drainage [4].

## Case presentation

A 74-year-old white Italian female presented to our clinic in October 2024 for a recurrent soft tissue infection. While having previously undergone a hysterectomy for benign pathology, the patient had no history of chronic illnesses or immunosuppression and was not on home medications.

**Fig. 1:**
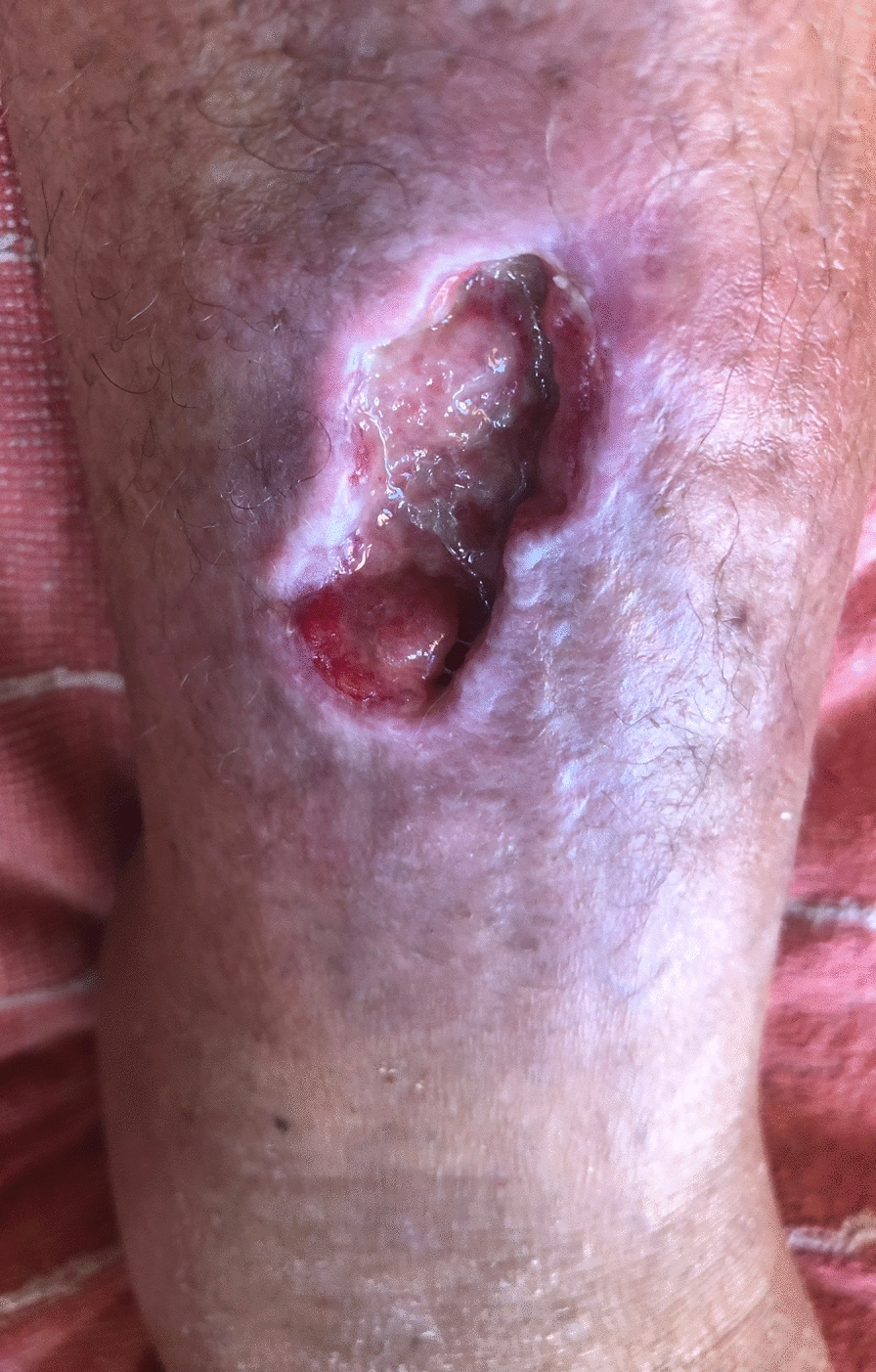
Wound 3 weeks after bathing activities in Valencia

In early July, she injured her right tibial crest by slipping and falling on rock, resulting in an open wound; 2 weeks later, after she bathed in shallow sea waters in Valencia, Spain, the lesion started to show symptoms of infection (Fig. 1). She was initially treated with oral amoxicillin/clavulanic acid at 875/125 mg every 8 hours for a total of 10 days. In addition, a wound debridement procedure was performed, and the collected purulent material tested positive for *Shewanella putrefaciens* with intermediate sensitivity (I) to quinolones and cephalosporins (cefepime, I, minimum inhibitory concentration (MIC) ≤ 1 μg/mL; ceftazidime, I, MIC ≤ 0.5 μg/mL; ciprofloxacin, I, MIC ≤ 0.25 μg/mL) and *Bacteroides fragilis* (multi-sensitive).

After 1 month, despite treatment, her symptoms persisted. Fever has never been reported; however, given the lack of wound healing and the persistence of signs of local inflammation, an ultrasound was performed, revealing a hypoechogenic area (8 × 11 × 24 mm) beneath the fascial plane without signs of local infiltration. Moreover, arterial and venous Doppler ultrasound of the lower limbs was performed, which ruled out significant stenosis or thrombosis.

**Fig. 2:**
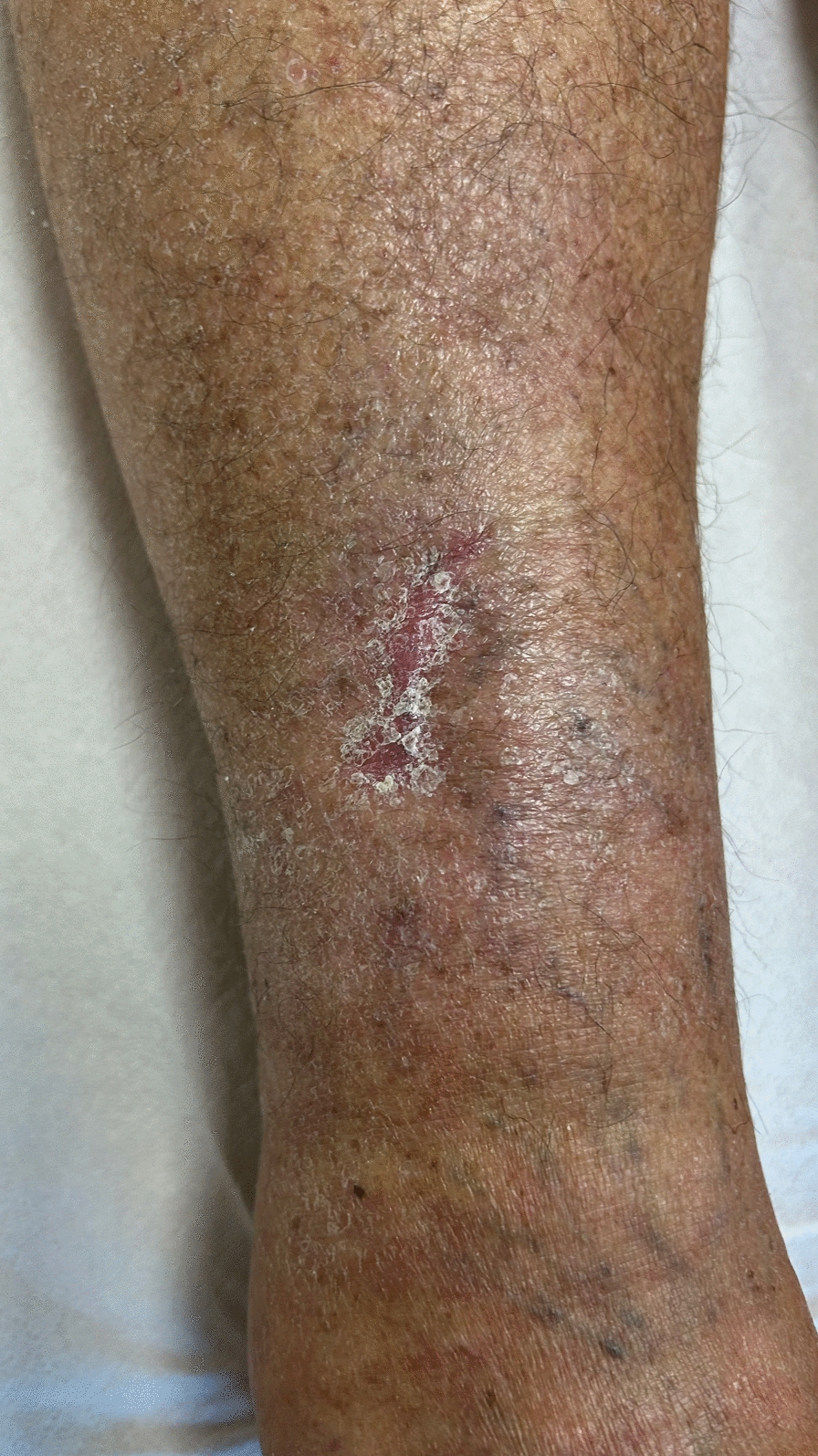
Wound 1 month after treatment with ciprofloxacin

Upon examination, the patient presented with persistent erythema and local swelling associated with hard edema of the right foot. Considering that the previous treatment was adequate for *Bacteroides fragilis*, a new course of antibiotic treatment was initiated with high-dose oral ciprofloxacin (750 mg every 12 hours) according to the antibiogram, in combination with a supplement for tendon protection. The patient self-withdrew from antibiotic therapy after 5 days for myalgia and insomnia. At treatment interruption, a follow-up ultrasound was performed: no significant fluid or fluid-debris collections were observed at the site of the reported lesion; there was a thickened and hypoechoic appearance of the immediately supra-fascial tissue, measuring ~ 2 mm in thickness, with a transverse extension of ~ 3–4 cm and a longitudinal extension of approximately 10 cm. At the site of the wound, this hypoechoic tissue reaches the skin surface through a short tract, with an extension of ~ 1–2 cm. The described tissue was interpreted as granulation tissue resulting from the healing of a localized inflammatory lesion. Over the following month, the lesion healed normally without signs of erythema or local inflammation (Fig. 2). The patient retained hard edema in the ankle, likely owing to lymphatic insufficiency.

## Discussion and conclusion

Potential risk factors for *Shewanella* spp. infections include chronic ulcers in the lower extremities and, more commonly, traumatic wounds exposed to seawater. Additionally, the bacterium predominantly affects immunocompromised individuals, such as those with severe renal failure undergoing dialysis, owing to the biofilm-producing capability of *Shewanella*, which makes peritoneal or hemodialysis catheters potential entry points for infection [4].

In our case, high-dose quinolone treatment led to complete resolution, confirming the pathogenicity and clinical relevance of the bacterium. Wastewater discharged from treatment plants and facilities into the sea, which contains antibiotics and heavy metals, may play a role in the development of antibiotic resistance. Climate change is directly linked to antibiotic resistance and the ability of bacteria to escape pathogens [4]. Recent studies have identified Shewanella strains that exhibit resistance to both heavy metals (such as cobalt, iron, and cadmium) and antibiotics, suggesting a potential co-resistance mechanism driven by selection pressure [5].

The rise in such infections is correlated with increasing coastal sea surface temperatures, highlighting an emerging public health risk in the context of global warming and a larger elderly population [6].

## Data Availability

The datasets generated and/or analyzed during the current study are included in this published article. The MICs were determined via the microdilution method according to the EUCAST guidelines (2024 version).
